# Dr. Ratier on the Hospital Practice of Paris

**Published:** 1825-10-01

**Authors:** 


### *Dr. Ratier on the Hospital-Practice of Paris.*

[Concluded from p. 111.]

### Hôpital des Enfans.

This establishment is exclusively appropriated to the treatment of young persons under sixteen years of age, whatever be their sex or disease. Drs. Jadelot and Guersent are the physicians, and give lectures on the complaints peculiar to children. M. Baffos, senior, has charge of the surgical wards, in which any thing very interesting seldom occurs. Individuals having chronic affections, scrofula, tetters, and itch, are placed in separate apartments from those suffering from acute diseases. Itchy patients are treated by the *brothers Mahon*, who have been authorized to make experiments under the inspection of the physicians of the establishment; and we are gravely informed by M. Ratier, that the results appear to be—" assez avantageux!" Here then is a picture of quackery doling out nostrums *by authority*, and superintended by men whose unhallowed heads had been crowned by the "Bennet d'Hippocrate!" O though greatest city of the GREAT NATION, how art thou degraded!

According to M. Ratier, the practice of Dr. Guersent is extremely simple and rational: he attaches great importance to the investigation of symptoms, and never employs "energetic medication" but with particular caution, being persuaded that Nature never has more resources than during the age when the organs have scarcely yet exercised their functions; *i. e.* in infancy and early youth. Observation of the sick children, and numerous researches in pathological anatomy, have convinced Dr. G. that inflammations form by far the greatest part of juvenile diseases: Dr. Jadelot seems to entertain similar sentiments: we give a summary of the treatment usually instituted by the last of these physicians.

Long before the revolution in medicine, (in France, of course,) relatively to essential fevers, Dr. Jadelot was led, by his experience in the management of infantile diseases, to ascribe a more precise seat to these affections; and now refers a great number of fevers to isolated or manifold inflammations within the abdomen, the chest, or the head: he is also satisfied that the proportion of these in the former cavity is by much the most considerable. He has no peculiar views regarding intermittent fever, but merely modifies the ordinary treatment according as he perceives, in the middle of the general disorder, a distinct alteration of function in one or more organs. In this way he employs, with incontestible success, a mixed practice, *i. e.* antiphlogistic remedies directed upon the parts that appear to be the seat of irritation, and, at the same time, bark alone, or united with camphor, in lavements. Almost every affection removed from the class of fevers, has been referred to that of the inflammations; and, in consequence of this, they have been studied with more care, and the treatment better adapted to their seat and their nature, has become shorter and more successful. Dr. J. we learn, was among the first to acquire positive knowledge regarding the phlegmasiæ, and especially those of the alimentary canal: he created, so to speak, new modes of recognising them, and arrived, by the sole inspection of the countenance, at an accuracy of diagnosis truly remarkable.

In small-pox Dr. J. is particularly solicitous about discovering the various affections which, either in a manifest or latent manner, may be complicated with the disease; and, by the existing circumstances, is guided in modifying his treatment. When a violent angina supervenes, he prescribes antiphlogistics and the most energetic derivatives, without regard to the period of the eruption: on symptoms of gastric irritation arising, he combats them by appropriate means, under the persuasion, that thereby the eruption will be much facilitated. In bad cases, he has recourse to excitants, and even to internal tonics, both by the mouth and by injection. During the period of suppuration, he recommends evacuating the matter contained in the pustules, especially when there are confluent groups, by opening them with the point of a lancet, or clipping off their tops with scissars, and wiping away the discharge with fine linen. In the convalescent state, he finds benefit in the use of tepid baths, either simple or emollient, for hastening the separation of the crusts and restoring the cutaneous functions.

Measles, more frequently than small-pox, presents a complication of inflammation, either "mucous or parenchymatous," of the thoracic organs; and this complication requires as much, or even more attention than the principal disease. In such cases, Dr. J. is in the habit of adding to the usual antiphlogistic remedies, warm hand-baths, made more active by an admixture of vinegar, common salt or powdered mustard, for the purpose of diminishing the pulmonary congestion. He ascribes a sudden suppression of the measly eruption to the development of inflammation in some other part of the economy; and he attacks this new phenomenon with a degree of energy proportioned to the danger to which the child is exposed. If the eruption still fail of re-appearing, he has recourse to vapour-baths, or dry frictions, or the excitement of irritating liniments. Dr. J. never hesitates in prescribing tonics and stimulants, both internally and externally, when the eruption becomes imperfect by reason of constitutional debility. He restricts convalescents to a very severe regimen; and, to such, very rarely exhibits purgatives: when these are requisite, the mildest are preferred.

Scarlet fever is treated by Dr. Jadelot nearly in the same way as measles: he recommends examining the throat assiduously, for the purpose of anticipating the first movements of the gangrenous angina, with which this kind of fever is liable to be complicated.

Whether it be consecutive to scarlet fever or manifests itself independently of any precursory disease, the gangrenous angina *(putrid sore-throat)* is regarded by Dr. Jadelot as being identic. On the first appearance of this affection, when it is usually inflammatory, he employs a purely antiphlogistic system of treatment: but, so soon as he observes broad whitish specks on the posterior surfaces of the mouth, accompanied with signs of general debility, he instantly directs the application of sinapisms to the feet, injections of the decoction of bark into the throat, cataplasms of rice-flour made with the bark decoction, and moistened with aromatic vinegar at the moment of being applied under the jaw, together with lavements, consisting of camphorated decoction of bark, fumigation of the throat with vinegar, and demulcents. He also promotes the action of the cataplasms of bark, by frictions with the ammoniated liniment. Sometimes, but very rarely, he has recourse to tonics, internally administered.

Inflammation of the mucous membrane of the respiratory passages is so much the more dangerous, says our author, as its seat is nearer the larynx; and Dr. Jadelot is announced as being the first who succeeded, "by an attentive examination of the patient," in discovering the precise seat of the malady. This, of course, is of immense importance with regard to the treatment, which ought to be conducted with an activity and promptitude proportionally great, according as the angina is laryngeal or tracheal, and still more so when the wind-pipe is universally inflamed. This treatment, suggested, no doubt, by the doctor's discovery, consists of leeches to the anterior surface of the neck, sinapisms on the lower extremities, laxative or purgative lavements, and emetics generally of ipecacuan in powder or syrup: he gives the latter to feeble, unexcitable patients, after having subdued the inflammatory symptoms by local bleeding; in such cases, he induces vomiting frequently and at short intervals.

Dr. J. considers croup as a kind of angina of the windpipe, the true angina having more violent symptoms, and being characterised by decided paroxysms, separated by distinct remissions. He admits different degrees of the disease according to its intensity, without changing his opinion of its nature. Abstraction of blood, with leeches and emetics, are his most common means of treating croup: vomiting alone often succeeds in repressing it in weak, pale, flabby children; but, in those of opposite constitutions, he invariably applies leeches, and encourages the subsequent discharge of blood, till such time as the patient becomes pale and the pulse feeble. If the bleeding after leeches be too soon stopped, there is a danger of the disease reviving, and thus requiring a repetition of the same means. After the bleeding, Dr. J. prescribes vomiting, to be several times repeated, at intervals of two or three hours; and this practice, says M. R. is followed with the greatest advantage, for the young ones are relieved by the effects of each emetic. When croup has past into its second stage without interference, and there is reason to suspect the presence of a "false membrane," the doctor still begins with leeching; but, so soon as the animals drop off, he instantly proceeds to induce vomiting; and, for this purpose, employs the "anticroupal potion," *(syrup of ipecacuan, oxymel of squills, and tartrite of antimony, in an infusion of rattlesnake-root,)* in graduated doses, at the end of every ten or fifteen minutes till vomiting supervenes. At the same time, he employs the action of derivatives on the skin and intestinal canal, and also endeavours to excite sneezing.

"When the progress of croup is very rapid, whether ought we to begin with bleeding or vomiting," is a question of M. Ratier's! Dr. Jadelot is of opinion that we should bleed first, if the child is robust and there are symptoms of congestion in the superior parts, (the head we presume.) On the contrary, we should commence with inducing vomiting; but afterwards have recourse to leeching, when the patient is pale, "comme aneantis," and there is little heat and fever. In a paragraph, which we do not profess to understand, it is said, that Dr. J. administers sulphate of potass in croup, especially when it is very inflammatory, and, sometimes, in the opposite circumstances: he varies the doses, but limits the quantity to half a drachm in the twenty-four hours.

From a multitude of observations, Dr. Jadelot has been led to conclude, that chin-cough consists in the inflammation of the bronchial tubes, by which ordinary catarrhs are distinguished, and that this is accompanied by a particular lesion of the nerves. Hence, he treats the disease with sanguineous and other depletions, using, at the same time, narcotics internally and applied to the chest, with derivatives, such as sinapisms, so modified as to produce slight redness, and frequently repeated. He likewise derives advantage from frictions of the arms and surface of the chest with acetic ether. His narcotic cataplasms are moistened with a drachm of laudanum, or a solution of the extract of opium: he obtains good effects from blisters, in the last stages of the disease.

In acute hydrocephalus, Dr. J. recognises a gastro-intestinal irritation which manifests itself at the outset of the disease, and, in one degree or other, precedes the development of the cerebral symptoms: he opposes this irritation with emollient applications over the abdomen, and topical bleedings. Subsequently, and when the head has become the principal seat of the affection, he directs his antiphlogistic means to this part, without, however, losing sight of the abdominal irritation. In the first stage, when there is much congestion of the brain, and before effusion has commenced, Dr. J. places ice on the head; but he advises the never having recourse to this proceeding till after local bleeding, and only when the patient lies immersed in a tepid bath. When symptoms of effusion in the brain are perceived, he institutes the use of external derivatives: a blister is placed on the nape, and the arms and lower limbs are rubbed with acetic ether or volatile and aromatic liniments. He employs mercurial frictions on the shaved head, (half a drachm of the ointment for each) and repeats them every 3, 4, or six hours, after carefully cleansing the surface with ammoniated liniment. In the mean time, he administers calomel by the mouth, in doses of 2, 3, or 4 grains, repeated four or five times in the hour: in fine, his ultimate resource ("le moyen extrême") is a very large blister over the head.

Such is an outline of Dr. Ratier's general observations on the French metropolitan hospitals: in this, we have been somewhat particular, with the view rather of affording the readers of this Journal an opportunity of appreciating for themselves the actual state of medicine in France, than with the hope of contributing any thing to their practical instruction. Our author concludes with this summary:—

"Whoever visits the hospitals of Paris, will observe, with satisfaction, that the practitioners who attend the sick in them emulate each other in zeal and talent, and substitute an analytic ('une marche analytique') and rational practice, for the subtle, and sometimes seducing, theories of the ancient physicians: he will find, in comparing their manner of acting, a harmony, an analogy, which constitutes a strong proof in favor of the existing system of medicine. In these institutions, the physiological doctrine, variously modified, but resting untouched in what concerns therapeutics, is almost universally adopted, either in an evident manner, or by a gradation easy to be followed. In general, much reserve is exercised in the employment of tonics, stimulants, and other '*remèdes perturbateurs*;' means doubtless very useful, but which have been singularly abused. Observation and experience have led back practitioners to '*la médécine expectante*,' (a most surprising result assuredly!) They have some confidence in the *conservative* efforts of Nature, and *polypharmacy* has given place, in many instances, to the judicious combination of *hygienic* agents. Nevertheless, they do not neglect the resources of the materia medica; and, by experiments repeated in many hospitals and by different practitioners, apply themselves to determine the properties of new substances, to verify those which the ancients had attributed to the remedies they have transmitted to us, and to ascertain their usefulness under new applications. In fine, instead of wasting time with composing learned, but too frequently worthless, dissertations, they multiply particular observations and necrotomical researches, which alone form the solid foundation of medical science."

Having essayed, we trust with generous courtesy, to accompany Dr. Ratier in his progress to this comfortable conclusion, we are very unwilling to disturb this gentleman's self complacency; but we must really take the indulgence of submitting just one observation on this part of his book; and it is this, that he might do well in making himself better acquainted with the improvements in medicine in other countries, especially in Great Britain; and we can assure him that, on his having done this, he will be more careful of giving such undue scope to the national "amour propre," and of exposing what ungenerous critics might denominate his ignorance, but which we shall call by the gentler name of inexperience, in ascribing as discoveries to his patrons, what had been discovered before these men were born, and in announcing modes of practice as important, which, in our estimation, are little better than mischievous puerilities.

II. PRACTICAL FORMULARY. This portion of Dr. Ratier's volume occupies 242 pages: it is a mere list of prescriptions, interspersed with brief and rather jejune remarks on the properties of those medicines whereof they are composed. Although this list, copious as it is, contains little that can be made available by a British practitioner, we propose to run over its different branches, and make such selections as shall appear worthy of a cursory notice. The subjects it includes are, *baths, cataplasms, collyria, fomentations, fumigations, gargles, injections,* (surgical) *lavements,* (clysters) *liniments, ointments, pills, potions, compound powders, and ptisans.* Let us attend to these, *seriatim.*

*Baths.* Under this head are arranged the domestic and medicinal, general and local baths, which last comprehend what is called "maniluves," and "douches," and affusion. Of these the most remarkable is the sulphureo-gelatinous bath, employed by M. Dupuytren: it consists of four ounces of sulphuret of potass, dissolved in two hundred pounds of water, with two pounds of white Flanders glue, melted in ten pounds of boiling water, added to the solution. This has all the advantages, we are told, of a simple solution of sulphuret of potass, without its inconveniences: it neither irritates, nor agitates, nor excites feverishness, and communicates unctuous and emollient qualities, which render it preferable to the ordinary sulphureous baths, in all cases where irritating effects are to be avoided.

*Cataplasms.* That used for promoting the resolution of inflammatory venereal tumours, is made by mixing one ounce of the sub-acetate of lead and half a drachm of the hydro-chlorite of ammonia, with four ounces of a common poultice. In the Hôtel Dieu, they employ, with much success, a cataplasm of powdered bark and slices of lemon in a poultice of linseed, for the cure of hospital-gangrene.

*Collyria.* The "collyre de Conrad" is powerfully resolvent, they say, as well as sedative: it results from dissolving one grain of the deuto-chloride of mercury, one scruple of gum tragacanth, and eighteen drops of liquid laudanum, in four ounces of rose-water. This collyrium is useful in obstinate ophthalmies, whether produced by the syphilitic vice or other ordinary causes.

*Fomentations.* Lotions are included under this head: of these we shall select three: the rest are good enough perhaps; but, being adapted to the "médécine expectante," they will be little esteemed by physicians in this country, where that practice is not fashionable. 1st. The *anti-psoric lotion* of M. Dupuytren, is prepared by adding four drachms of dilute sulphuric acid to a pound of water, holding four ounces of sulphuret of potass in solution. In cases of itch, the parts covered with pimples are fomented, two or three times in the day, with this lotion: its effects are assisted by occasional repetitions of the common bath. 2nd. M. Manry's *mercurial lotion* is in general use for the cure of itch, and has been found very efficacious in the prurigo formicans and pedicularis: it does not excite salivation, we are assured, as some persons have erroneously stated: the addition of one drachm of camphor to each pound of the lotion, is said to *correct* its irritating action. Its proportions are, two ounces of crude mercury, four of nitric acid, and ten pounds of distilled water: the mercury is first dissolved in the acid, and the solution extended by adding the water: the dose is half an ounce, with which the parts are washed every morning and evening. 3d. Professor Alibert's lotion, for similar purposes, is a compound preparation: he directs, first, two ounces of sulphuret of potass to be dissolved in one pound of river (soft) water, and then two ounces of the hydro-chloric acid to be diffused into one pound of distilled water: an ounce of each liquor is poured into four ounces of warm water, and applied as a lotion to the parts with a sponge. These, as remedies for several cutaneous eruptions, are described as having few inconveniences, and often proving very beneficial.

*Fumigations.* Those most frequently employed, impart their effects in the vapours of sulphur, cinnabar, mercury, alcohol, and aromatic substances. Of all others, the fumigation with gas of chlorine is the most powerful; but, we agree with M. Ratier, in suspecting that the advantages derived from such processes have perhaps been exaggerated; and, in itch, for instance, their application is frequently unprofitable.

*Gargles.* In regard to these, our author premises a remark —which, in our mind, is judicious, and merits attention: it is this, not to agitate the fluid of a gargle in the back parts of the mouth, as is usually done, but to leave it in apposition with the diseased parts by holding back the head as far as possible. An active mercurial gargle is made by dissolving two grains of the deuto-chloride of mercury in four ounces of distilled water, and then adding half an ounce of syrup or honey: it is employed in cases of angina, accompanied with syphilitic ulcers of the pharynx and velum palati; especially when such ulcers are obstinate.

*Lavements.* Among these, the *antisyphilitic and tonic* are the most remarkable. The former consists of two grains of the deuto-chloride of mercury dissolved in two ounces of distilled water, and one pound of the decoction of linseed, into which the mercurial solution is diffused. In the use of this, an attempt is made to introduce the muriate of quicksilver into the lower intestines, in cases where the stomach cannot bear that medicine. It is, however, an uncertain remedy, and ought not to be exhibited till other means have failed: in France, forgetting that mercury removes local disease only by its action on the general system, they use it when the rectum has become the seat of syphilitic symptoms. As a tonic clyster, in cases of putrid fever, they put an ounce of bark into a pound of water, with a view to stimulate the intestinal canal; to the preceding, a scruple of camphor, or half an ounce of muriatic acid, or a scruple of laudanum, may be added according to circumstances.

*Liniments.* That for chilblains contains at least a sufficiency of ingredients. For its composition, two ounces of olive-oil, one drachm of balsam of Peru, two drachms of spermaceti, two of white wax, and two of hydro-chloric acid, with six drachms of water are intimately mingled together; we have not taken an opportunity of reducing it to the test of experiment, but feel disposed to regard this as both an elegant and efficacious preparation. For cutaneous diseases, Dr. Jadelot employs a liniment, combining sulphuret of potass, white soap, and vegetable oil; but he ordinarily accompanies it with simple or sulphureous baths.

*Ointments.* The most remarkable of these are the mercurial, and such as contain iodine. In one of the former are combined, three ounces of axunge, half an ounce of the red oxide of mercury, the same quantity of crystallised acetate of lead, the same of sulphate of alum, the same of oxide of zinc, and fifty grains of the deuto-chloride of mercury: it is used in cases of obstinate chronic ophthalmy, having a connexion with the scrofulous constitution. This may perhaps be a suitable remedy; but in our mind, there are not many modifications of scrofula which derive advantage from the administration of mercury. Antimonial ointment (a drachm of the tartrite of antimony to an ounce of purified lard) is employed in French practice, under the name of "la Pommade d'Authenrieth," as an active derivative in a great number of diseases, especially chincough, and in general all inveterate affections of the chest. M. Biett has introduced four "pommades" containing iodine, into the wards of the "Hôpital St. Louis," and regards them as very manageable as well as efficacious applications. For one, he mixes fifteen grains of the deuto-iodate of mercury and twenty drops of the essence of bergamot, with two ounces of prepared lard: another contains half a drachm of the proto-iodate of mercury, an ounce and a half of lard, and fifteen drops of the essence of bergamot: the third, is made with sixteen grains of the cyanate of mercury, an ounce of axunge, and fifteen drops of the essence of lemon: and the fourth includes sixteen grains of the hydro-cyanate of mercury, one ounce of lard, and ten drops of the essence of bergamot. With the first, M. Biett cures, or greatly modifies venereal tubercles, and the chronic ulcers arising from the syphilitic constitution; but its intense activity requires to be vigilantly superintended. The second is milder, though still a very efficacious remedy: very large venereal ulcers, which had resisted a multitude of other means, have been found to cicatrise under the application of this "pommade:" this, as well as the former, sometimes induces salivation. M. Biett began his experiments with the combinations of mercury and iodine, in 1821: at first, he prescribed the proto-iodate in small doses; but he soon ascertained that, in the proportion of $\frac{1}{24}$ in an unctuous recipient, it might be adopted in the greatest number of cases. The deuto-iodate being more active than the deuto-chlorate of mercury by itself, produced in several patients a very intense erysipelatous inflammation of the parts over which it was applied: this, however, was of short duration. After many trials, M. B. fixed on the preceding formula; but even this, he says, will require being varied according to the susceptibility of individuals. Iodine and its combinations certainly offer new and powerful resources in the healing art, and merit the attention of experimental physicians.

*Pills.* Under this head are also included boluses and electuaries: they are sufficiently various; some of them sufficiently simple; others sufficiently complicated. It is somewhat surprising to find the French physicians, who seem to entertain an absolute horror of our "heroic" remedies, prescribing so freely the internal use of the muriate of quicksilver, both in pills and other modes of composition. In the "Saint Louis," they have three kinds of mercurial pills, one of which is rather singular, but perhaps not the less useful: three ounces of the strongest mercurial ointment, two of medicinal soap, and two and a half of starch are compounded and made into pills of four grains each: the dose is one, sometimes two, in the evening and morning, as the basis of the anti-syphilitic treatment. M. Dubois' "depurative" pills are made by mixing half a drachm of calomel, eighteen grains of the extract of opium, four drachms of the extract of hemlock, with a sufficiency of the syrup of marsh-mallows, and dividing the mass into thirty-six pills. He prescribes these in different organic affections, whether glandular or visceral; but he regards them, not so much as curative remedies, as means of allaying the distresses whereby persons having such diseases are afflicted: two, at first, are a dose which is gradually increased. From observation of its effects, under a modified form *(ipecacuan instead of antimony)* we willingly bear testimony to the usefulness of an aperient pill employed in the Maison de Santé: it is obtained by compounding one scruple of socotrine aloes, three grains of tartrite of antimony, and half a drachm of extract of gentian, and dividing the mass into twenty pills: one or two of these act very beneficially in removing habitual constipation.

We are inclined to think that Dr. Duncan, in the Edinburgh Dispensatory, has expressed himself in a manner not strictly philosophical, respecting the medical employment of lead. "The internal use of acetate of lead," he says, "notwithstanding the encomiums some have been rash enough to bestow upon it, is entirely to be rejected." Now, it may be asked, how shall we reconcile this dogma with the experience of Drs. Duméril and Fouquier who administer the proscribed remedy in the wards of two large hospitals, and find their practical intentions answered by its effects? The former makes four grains of the acetate of lead, one grain of opium, and one scruple of the extract of liquorice into sixteen pills, which are exhibited in chronic and colliquative diarrhœa, for the purpose of diminishing the alvine evacuations. Dr. Ratier forgets to tell us the dose of these pills; but he states unreservedly that "the salt of lead appears to be *too weak*." M. Fouquier directs a drachm of the acetate of lead, and an equal quantity of marsh-mallow in powder, to be made with a sufficiency of syrup into thirty-six pills, of which the dose varies from four to a dozen in the day, at regular intervals. They are prescribed, says Dr. R. for moderating the perspirations that debilitate phthisical patients; and they fulfil this object in a satisfactory manner, without producing the accidents some persons would lead us to apprehend. Ordinarily, eight or ten pills are sufficient for obtaining the desired effect: it is proper that they be taken about the time when the return of perspiration approaches; and this, they are often found to repress *as if by enchantment*: *i. e.* in sober terms, the medicine soon accomplishes the ends for which it is prescribed. In such cases, we ourselves are in the habit of administering *internally* the solution of acetate of zinc, and generally have found it to act, not by enchantment certainly, but with very manifest advantage.

*Potions.* Juleps and fluid mixtures of all kinds are arranged by our author under this class, which occupies nearly sixty pages of his book. Most of these would be regarded as very inefficacious remedies in this country; but this is no reason, we readily admit, why they should not be exceedingly useful in different circumstances, and in a different climate: we cannot, therefore, agree with those who would depreciate the continental modes of practice, because the agents employed in it would be useless, if not pernicious to the sick in these islands. We offer a few specimens of the Parisian potions.

In the Hôtel Dieu, they give a tonic potion composed of two drachms of the extract of bark, half a drachm of extract of opium, two ounces and a half of the vinous syrup of bark, four ounces of distilled peppermint-water, and an equal proportion of water distilled from canella alba. Professor Dupuytren exhibits this mixture, in divided doses, in diseases where there is great prostration of strength unaccompanied with gastro-intestinal inflammation. With reference to the use of this remedy, M. Ratier makes an observation the perfect simplicity of which could not fail exciting mirth, did we not believe him to be quite serious. "It ought to be observed," he says, "that this celebrated practitioner (M. Dupuytren), even before the appearance of the new physiological doctrine, had directed his attention to the general abuse of tonics, and become extremely circumspect in his use of them: he very rarely prescribes them, and never except in cases where the necessity is evident." In the same hospital a somewhat elegant diuretic mixture is in repute: it is formed by dissolving an ounce of acetate of potass in a pound of white wine: it is administered in dropsies, both of the cellular texture and of the serous cavities: the solvent is understood to promote its exciting qualities; but it should never be exhibited when there is irritation of the bowels. Professor Chaussier employs an antisyphilitic liquor, made by dissolving eight grains of the cyanate of mercury in a pound of distilled water: it is exhibited in graduated doses, varied in strength to suit the predominating circumstances. Of all the Parisian potions, in fine, *the eau bénite* is the most active: it is a solution of six grains of tartrite of antimony in eight ounces of water. This, says M. Ratier, is an emetic of great energy, and requires being used with the utmost reserve.

*Compound Powders.* The aromatic powder of M. Dupuytren contains pulverised thyme, sage, and rosemary, of each four ounces, hydro-chlorate of ammonia and camphor, of each a scruple, all intimately compounded: it is sprinkled on mortifying parts, and those affected with hospital-gangrene. In the "Saint-Antoine," they substitute for Plummer's pills, a powder combining the "orangé" sulphuretted oxide of antimony, and the protochloride of mercury, of each a scruple, rubbed into half a drachm of sugar, in doses of five or six grains, night and morning; it is administered for venereal and scrofulous affections. By mixing half a drachm of the white oxide of arsenic, one ounce of Dutch vermilion (red sulphuret of quicksilver,) and half an ounce of powdered dragon's blood, M. Dubois forms a preparation which promotes the cicatrization of chancrous ulcers. Having fretted the parts so as to cause them to bleed, he covers them with a paste made of this powder and saliva, and then overlays all with a piece of cobweb; at the end of a few days, the eschar is detached, and leaves a florid surface disposed, in general, to send out healthy granulations.

*Ptisans.* Including decoctions, infusions and "lemonades;" we have ninety-one compositions of this kind, which, says the author of the "Formulaire," contain in general "qu' une très petite portion de substances médicamenteuses." As we implicitly adopt this opinion, we shall not extend this article with a notice of more than one, which is the celebrated "Tisane de Feltz:" for preparing it, they put three ounces of sliced sarsaparilla, half an ounce of isinglass, and four ounces of pulverised antimony, into six pounds of water, and reduce the liquid one half by boiling. This ptisan, it seems, is exhibited with advantage for inveterate venereal affections, and such as obstinately resist the mercurial treatment: the dose is a pound and a half in the day, taken in three equal portions. According to M. Ratier's report, it is a remedy extremely advantageous; and, in a few days produces a visible improvement of the patient's condition.

III. Particular Remedies. Passing M. Chaussier's watery infusion of opium, two vermifuge, and one antisyphilitic remedies, which have nothing particular in them, we present our readers with the *resolvent pouch* ("sachet") of M. Dumeril. He puts sulphate of lime, sulphate of iron, and hydrochlorate of ammonia, of each a drachm, into a wadding ("*ouatte*") of cotton, enveloped with black silk on the outside and with thin muslin on the surface next the wearer's skin. This pouch, in the form of a collar, is worn by persons having goitrous and other tumours arising from morbid enlargement of the thyroid gland.—Curiosity is generally irascible; and this, we suppose, it is that makes us so angry with Dr. Ratier for leaving us quite in the dark respecting the effects of this picturesque remedy. M. Corvisart's bitter diuretic wine, and the bitter elixir of M. Dubois are good enough in their way; but we prefer being particular in our account of M. Dupuytren's treatment of scrofulous diseases.

*Scrofula.* Professor Dupuytren informs Dr. Ratier that his mode of treating such diseases is altogether different from that in general use; and that he was led by the results of his anatomical and physiological observations on the nature and progress of scrofula, to renounce the "vulgar method." Whatever be its seat or its varieties, the scrofulous affection, in his mind, has three distinct stages in its course. In the first, the disease is in some degree inert, and only manifests itself by the characters peculiar to the lymphatic constitution, and by a disorder, more or less difficult to be appreciated, in the actions of the affected parts. During this stage, he employs the means best adapted to strengthen the constitution and, consequently, to "*resolve*" the disease. At the same time, he carefully avoids what is irritating, agitating, or even heating, such as elixirs, anti-scorbutic syrups, and all spirituous mixtures, with every stimulant likely to hurry the disease from the inert into an inflammatory state. It is, he thinks, in the second stage particularly, which is always characterised by excitement, fever, local pain and enlargement, and "sanguineous exhalations," that the abuse of these "incendiary remedies" does more mischief than the scrofulous affection itself. Without paying regard to its "*presumed nature,*" M. Dupuytren treats scrofula, in the second stage, with venesection, leeching, diet and demulcents; and, by these means, has often arrested its progress and prevented its consequences, such as caries of bones, gibbosity, spontaneous luxations, suppuration, with disorder and destruction of organs. So soon as suppuration is established and the discharge readily escapes externally, he considers the disease as having returned to the nearly inert state which constitutes its primary stage, and resumes the use of remedies adapted to modify and invigorate the constitution; but, in doing this, is always solicitous of withholding whatever may tend to occasion irritation, agitation, insomnolency, loss of appetite, or fever. For such reasons, he abstains, throughout this third stage, from exhibiting vinous, alcoholic, alkaline, and other analogous preparations: he prescribes watery infusions of gentian, cinchona, and sinarouba, being persuaded that they contain all that is tonic in these substances, and are exempted from every thing irritating, both in their principle and vehicle: he increases their strength according to the patient's age and sex, and the kind, seat and character of the scrofulous affection: he suspends, in fine, even the administration of these, as soon as he perceives in them the slightest cause of excitement.

*Opacities of the Cornea.* These, under the hands of M. Dupuytren, yield to the following treatment; a general bleeding when the irritation is great; or leeches to the temples, when moderate;—a flat seton passing for several inches under the skin in the neck;—and an impalpable powder, composed of prepared tutty, candied sugar, and calomel, in equal proportions, blown into the eye through the barrel of a quill. When there is no disease of the eye-lids, nor inflammation, nor irritation of the conjunctiva, this powder alone is sufficient, and in a short time removes albugineous specks: old, thick, large opacities generally require a month or six weeks treatment; but, very frequently, such as have occupied nearly all the cornea and completely covered the pupil will, by the Professor's management, disappear in a few months. Our readers will be pleased to hold in recollection that, throughout this article, we are endeavouring rather to furnish them with historical facts in medicine, than with contributions to their professional knowledge.

*Phagedenic Tetters.* M. Dupuytren, in giving an account of the powder used by himself for phagedenic and eroding tetters, introduces the subject with this lamentation. There is no physician, he says, who has treated this disease, but may have found from sad experience, the inefficacy of anti-dartrous, anti-scrofulous, anti-venereal, and other remedies employed for this cruel malady, according to its different appearances, and the nature ascribed to it. Every body knows, he adds, that in spite of all these remedies, the phagedenic tetter still continues to erode and destroy the nose, the cheeks, lips, eyelids, ears, and temples, which parts it seizes, as it were, by preference. Fire itself appears to cause irritation (it would be singular if it did not,) as well as the arsenical paste: these agents, moreover, destroy the sound substances to which they are applied, and in this way, increase the deformity. Knowing all these things, the Professor laid himself out to discover a more certain application, and soon satisfied his own mind, that the disease might be perfectly cured by a powder, consisting of 199 parts of impalpable calomel, and 1 of arsenious acid. When the parts have ulcerated, they are moistened and sprinkled with the powder so as to form a crust, for the removal of which a poultice sometimes becomes necessary: should the tetter be actually covered with an unsound cicatrix, this must be destroyed, and in twenty-four hours, when the bleeding shall have ceased, the powder may be applied: it will be more adhesive when suspended in a solution of gum arabic, or incorporated with a mild ointment; but, in such cases, the acid ought to be increased by one or two one-hundredth parts additional. In all cases, the crust however produced, should be left to undergo a spontaneous separation, which usually occurs in eight or ten days: the same process is to be repeated till the cure is complete.

*Syphilis.* Some remarks well meriting consideration, are prefixed to M. Dupuytren's description of his mode of treating venereal complaints. Syphilis, we are informed, is very often complicated with surgical diseases: itself alone produces a great number. Hence results the necessity of combining antisyphilitic with chirurgical remedies in the method of curing these complicated affections. In such instances, it is in vain for any one to attempt treating separately the principal disease and its syphilitic complication: the selection of means is, therefore, of the greatest importance. Metallic mercury (quicksilver) reduced to the state of an oxide by trituration with lard, and applied by friction, cannot be administered with convenience in general hospitals and large wards, which seldom enjoy the warmth of temperature that is favourable to cutaneous absorption; besides, this preparation has the inconvenience of becoming rancid on the surface of the skin, (has the occurrence of such a phenomenon been observed in this country?) and, in consequence, diffuses an unsalutary odour, injurious alike to the patient and the other inmates of his ward. Sublimate (muriate of mercury) administered internally, in a concentrated or copious diffused solution, has other inconveniences: in the former state, it irritates and inflames the stomach, *and also the lungs* (this affords a hint to those who may not yet have particularly considered the actions of mercury when exhibited for disease of the respiratory organs;) in copious solution, it is apt to sustain a decomposition; a precipitate descends, and the medicine loses its specific virtues; when left, moreover, as it necessarily often is, to the management of the sick themselves, its administration is generally very irregular. That form of syphilis, in fine, which is usually complicated with surgical diseases, is almost always of long standing, inveterate, constitutional, so to speak, and not seldom characterised by nocturnal aggravations and other unpromising symptoms. Consideration of these things appears to have directed M. Dupuytren in his choice of the remedies for such complicated affections. Hence, his practice unites sudorifics, mercurials and anodynes, "médicamens dont la combinaison constitue l'ensemble le plus propre à faire cesser les douleurs, et à combattre le vice venerien le plus ancien et le plus rebelle."

Professor D. prescribes his sudorific ptisans and syrups first, and then the sublimate and opium formed into pills. The former is made by putting China-root *(similax China,)* sarsaparilla and guaiac, of each half an ounce, into two pounds of water, and evaporating one third of the fluid by boiling. This quantity is used every day in divided doses; but to the first in the morning, and the last in the evening, two ounces of a sudorific syrup are added. The pills, each of which contains two grains of the extract of guaiac, half a grain of the watery extract of opium, and one eighth of a grain of the deuto-chlorate of mercury, are given in the morning, at mid-day, and at night, an hour at least before meals: a single pill constitutes a dose; and the effects of this twofold treatment are promoted by, what is here denominated, a moderate diet. We do not reckon any of our readers so simple, or so ill informed as to require being told that these *new* discoveries of M. Dupuytren's are just about *two centuries and a half old*: those among them who are *very young students* can readily satisfy themselves on this subject by turning up the common dispensatories and class-books of the materia medica, at the words, sarsaparilla, guaiac, china-root, and mercury. We do not, however, mean to dispute the efficacy of these drugs: what a wise man states to be fact, we are at all times ready to admit as being a fact, till a wiser man affords us reason for changing this admission into doubt or disbelief.

M. Dupuytren does not usually employ any local antisyphilitic applications. On this account, the local symptoms become, in some sort, "*the thermometer*" of the efficacy of the internal remedies, and almost always disappear in no long time after the latter are put in use. This is the order of their disappearance; the night-pains, in eight or ten days; the venereal character of wounds and ulcers, in "some weeks;" indurations, excrescences, and even ulcers themselves, in a month or six weeks; and the exostoses, last of all, but when we are not told. Ordinary surgeons generally bring themselves to believe that venereal affections have been radically cured when their symptoms have disappeared; but Professor D. is persuaded that they are then only palliated, in by far the greater number of cases: and he ascribes to treatment conducted on such principles the spontaneous relapses which so often recur in hospital-practice. On this account, he has made it a general rule, in treating constitutional venereal diseases, to continue the administration of curative means, after the total disappearance of all the symptoms, during a space of time equal to that which was required for dissipating these symptoms. It is then, and then only, the Professor assures us, that the cause and the effects are alike well cured, and without any danger of relapse.

*Inflammation of the Retina.*—Depression is the only method employed in the Hôtel Dieu, in operating for cataract. One of the most common and most serious consequences of this method, as well as that by extraction, is inflammation of the retina, "*denominated inflammation of the iris, or iritis, by those who are more struck by the apparent symptoms than by the cause and true seat of the disease.*" This affection, says M. Dupuytren, whose sentiments these are, has for its results—long and obstinate pain of the head and eye, contraction of the pupil, turbidness of the aqueous and vitreous humors, redness of the conjunctiva, incessant flow of acrid tears, intolerance of light, strong contraction of the orbicular muscles, formation, behind the pupil, of an accidental fibrous pellicle, to which the iris ordinarily becomes adherent, and produces blindness: this last, however, may be remedied, even at the end of some months, by destroying or displacing this pellicle with a cataract-needle.

Inflammation of the retina likewise very often attacks children affected with scrofula, and, in them, presents the same train of symptoms, with a still greater intolerance of light, and a contraction of the orbicular muscles of the eyelids. It obliges the little patients to seek the darkest places, and to press the eyelids so forcibly together as to make the inferior pass under the superior, and thus bring the hairs of the former in contact with the lining of the latter, and thereby increase the pain and danger from the disease.

No doubt, says Professor D. venesection, leechings, demulcents, and derivatives, such as setons and purgatives, are indicated, and useful in such cases; but experience has so often demonstrated their inefficiency, that he has felt himself obliged to look out for other remedies. That which he has exhibited with most advantage, during the last ten years, is the internal use of the *atropha belladonna*, both in powder and extract. Of the former, he gives three, four, eight, twelve, or a still greater number of grains, for a dose; of the latter, one, two, three, or more grains, make a dose; and the *dose*, both of powder and extract, is divided into six *doses*, one of which is taken at the end of every two hours. To prevent the narcotic influences, ("le narcotisme") whether local or general, which these medicines are apt to impart, he is in the habit of accompanying them with the use of artificial Seltz' water. It is quite useless, he concludes, to say, that the exhibition of bitters, antiscorbutics, and antiscrofulous remedies, so prodigally administered during the last twenty-five years, tend directly to induce and aggravate this kind of inflammation in children: but the principles of M. Dupuytren, says M. Dupuytren himself, make it sufficiently evident that he is not inclined to employ such means in this sort of inflammation, although it may originally arise from a scrofulous affection.

M. Dupuytren is the most eminent surgeon in France, perhaps on the continent: his practice is bold; in many instances rash and unreasonable: his practical opinions are generally announced with a peculiar air of confidence, which contributes to make them the more impressive: hence, with the unthinking and illiterate, they are received as oracles, and regarded as being altogether unparalelled both in originality and excellence. In this part of the world, however, it would be a mere waste of words to dispute either the one or the other; but, from the examples transferred to these pages, from communications by the Professor himself to Dr. Ratier, it must be manifest, even to the "barbarians of Britain," that old Dioscorides, who wrote and practised only eighteen hundred years since, was perfectly "au fait" with many of the *new discoveries* which M. Dupuytren blushes not to promulgate as his own, with a magnificence of gravity that is really ludicrous.

Dr. Ratier occupies the remainder of this section with short and, as we think, uninteresting notices of Fowler's solution, and other preparations of arsenic, a particular mode of treating the colica pictonum, on the nux vomica, strychnine, morphine, quinine, laurel-water, hydrocyanic (prussic) acid, iodine, nitrate of silver, henbane, belladonna, nightshade, lactuca virosa, (which yields the lactucarium of Dr. Duncan) stramonium, and rhus radicans, which M. Fouquier prescribes in paralytic cases, and M. Lerminier in dropsies of the cellular texture, connected with disease of the heart. Passing all the rest, we find ourselves induced, as well by its "*bizzarrerie*" as by the assurance of its success, to furnish the readers of our Journal with a condensed view of that mode of treating the colica pictonum, which has obtained the designation of "*traitement des Pères de la Charité.*"

On the first day, they exhibit a purgative lavement, prepared by boiling half an ounce of senna leaves in a pound of boiling water, and then adding half an ounce of the sulphate of soda and four ounces of emetic wine to the strained decoction. During the day, a drink is given in divided proportions: it is made by dissolving an ounce of sulphate of magnesia and three grains of tartrite of antimony in two pounds of cinnamon-water, to which an ounce of syrup of buckthorn is sometimes added. At five o'clock in the evening, an anodyne lavement is administered: the ingredients of this are, six ounces of oil of walnuts, suspended in twelve ounces of port wine. At eight, this is followed by a bolus, containing one grain of opium in a drachm of treacle. On the second day, the "*eau benite*" is prescribed as a vomit: the ingredients of this blessed potion are, six grains of emetic tartar, dissolved in eight ounces of tepid water: the patient takes this in two portions, and, in an hour, drinks freely of tepid water to promote its effects. When these have subsided, he receives, during the rest of the day, draughts of a sudorific ptisan, formed by putting guaiac, china-root, and sarsaparilla, of each a drachm, into two pounds of water, and boiling this down to one; an ounce of sassafras and half that quantity of liquorice are then added, and the mixture gently boiled and strained. At night, the anodyne lavement and bolus of opium and treacle complete the second day's discipline. The third morning begins with a diaphoretic laxative potion, administered in four doses, and, throughout the day, the sudorific ptisan in regulated draughts: at four, P.M. the purgative lavement; at six, the anodyne lavement; and, at eight, the opiate bolus. A purgative potion (half an ounce of sulphate of soda, a drachm of powdered jalap, and an ounce of syrup of buckthorn, in six ounces of the infusion of senna) introduces the fourth day's operations; and its action is assisted by "*du bouillon aux herbes.*" The patient drinks the sudorific ptisan through the day; at five, P.M. he gets the anodyne lavement; and, at eight, the bolus of treacle and opium. All the fifth day is occupied with draughts of the sudorific laxative ptisan: at four, P.M. comes the purgative lavement: at six, the anodyne; and, at eight, the opium and molasses; with which, as we suppose, the cure is consummated.

Such is the treatment, says Dr. Ratier, which, in spite of its "bizarre" composition, accomplishes a great number of cures, and which many physicians have thought it their duty to adopt with "*un religieux scrupule.*" *Pasques-Dieu!* as Louis XI. would have said, but these gentlemen must possess very tender consciences: the thing, however, is a rare one, and wise heads have, ere this, been led as far astray by pure curiosity. Dr. Lerminier follows the practice implicitly; but Dr. Fouquier employs it with considerable modification: he rejects all opiates, and, instead of an emetic, prescribes an emeto-cathartic, which may be three times reiterated. With him, to restore and establish regular alvine evacuation, constitutes the principal indication. He regards the means that are capable of overcoming the obstinate constipation, as the best for alleviating the rending pains which the patients experience.

We have thus, in two short articles, endeavoured to lay before our professional brethren, what we conceive to be an impartial and rather comprehensive view of French medical practice. In a happier climate than our own; with corporeal and intellectual temperaments different from our own; and with modes of life altogether unlike our own, such practices may prove sufficiently efficacious. That they are productive of the results ascribed to them, we feel not the smallest inclination to dispute; with us, however, they would not only be found unprofitable, but, in numerous instances, absolutely pernicious. From this exhibition of facts, then, we would deduce the important lesson—a lesson most worthy the attention of our countrymen—that, although by reason of facilities arising out of the moral constitution of the French people, our British students may find advantage in resorting to the Parisian schools for the purpose of more conveniently investigating some branches of demonstrative, physiological and pathological anatomy; yet, in all that regards the true objects of medical science—the relief or removal of human suffering, they will not only obtain little knowledge that shall be available at the bedsides of the sick in their native land, but they may incur the risk of imbibing notions that shall dispose them to become inefficient, and, by necessary consequences, very dangerous practitioners.

